# CTU findings of duplex kidney in kidney: A rare duplicated renal malformation

**DOI:** 10.1515/med-2021-0271

**Published:** 2021-04-19

**Authors:** Nanai Xie, Xu Huang, Jie Zhou, Heng Zhang, Wanling Ma

**Affiliations:** Department of Radiology, Longgang District People’s Hospital of Shenzhen & The Third Affiliated Hospital (Provisional) of The Chinese University of Hong Kong, Shenzhen, Guangdong Province, 518172, China

**Keywords:** kidney, urinary tract, computed tomography urography, duplex kidney, intrarenal kidney

## Abstract

Duplex kidney is a common congenital malformation appeared as duplication of pelvis and ureter. However, renal duplication within sinus renalis is an extremely rare variation of the renal collecting system. In this study, we report a case of an asymptomatic kidney disease in a 33-year-old man, who demonstrates abnormal echo of renal sinus anomaly discovered incidentally in ultrasound examination. Computed tomography urography (CTU) exhibited the other small duplex kidney located in renal sinus. In the excretory phase images, the contrast medium within its small renal pelvis could be seen to flow into the right major renal calices. This case exhibited a very rare anatomical variation of duplicated renal malformation.

## 1 Introduction

The renal duplication is a common renal malformation with an incidence of approximately 0.8% [1,2]. It usually appears as a renal unit comprised of two renal pelvis and kidney systems [3]. This malformation is characterized by incomplete fusion of upper and lower kidney moieties accompanied by complete or incomplete duplications of pyeloureteral [4].

However, duplicated renal malformation located in sinus renalis is an extremely rare anatomical variation. To the best of our knowledge, there is rare report concerning this malformation. In this study, we reported an extremely rare renal duplication: another little duplex kidney located in renal sinus. It is generally asymptomatic or presents with non-specific symptoms, and thus missed easily by clinician. On the plain computed tomography (CT) images, the abnormal renal shaped mass in the renal sinus is very easily misdiagnosed as the renal pelvis mass. Moreover, distinguishing it from renal columnar hypertrophy is very difficult during arterial and venous phases of the enhancement because of its similar enhancement to kidney. However, the CT pyelographic phase images demonstrate clearly that tubular contrast medium from the renal shaped mass merges into the renal pelvis, which verifies the existence of the duplicated renal pelvis.

## 2 Case report

**Figure 1:**
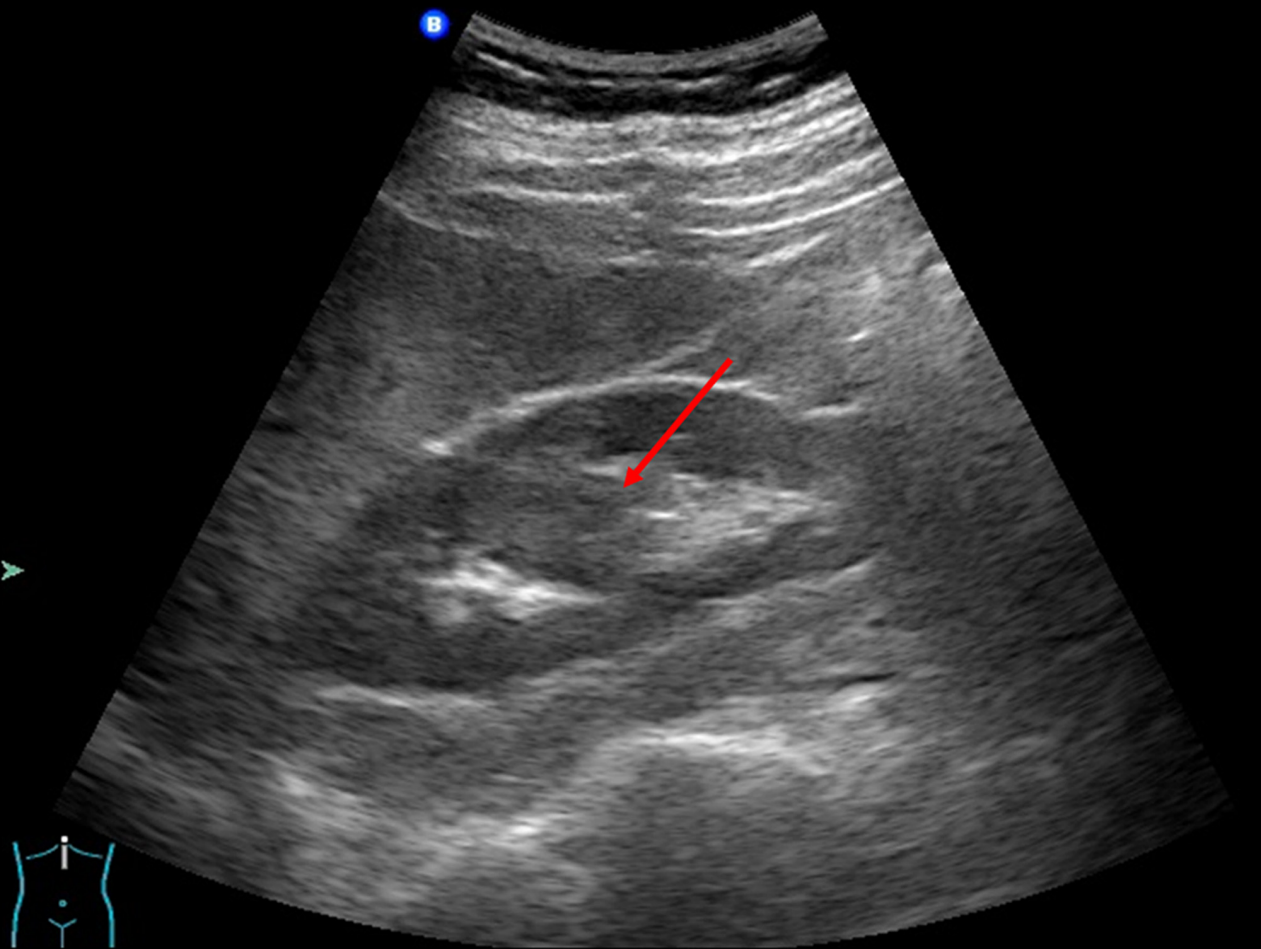
Ultrasound showed a patchy hypoechoic area (red arrow) within the right renal sinus, with slightly irregular shape, clear boundary, the similar echo of renal cortex, and enhanced echo in the rear.

Incidentally, a 33-year-old man was diagnosed as hypertrophied column of Bertin by ultrasound, which demonstrated abnormal echo mass in renal sinus (Figure 1). The patient was asymptomatic. All laboratory results and physical examinations were normal. No palpable mass and percussion pain were found in bilateral renal areas.

**Figure 2:**
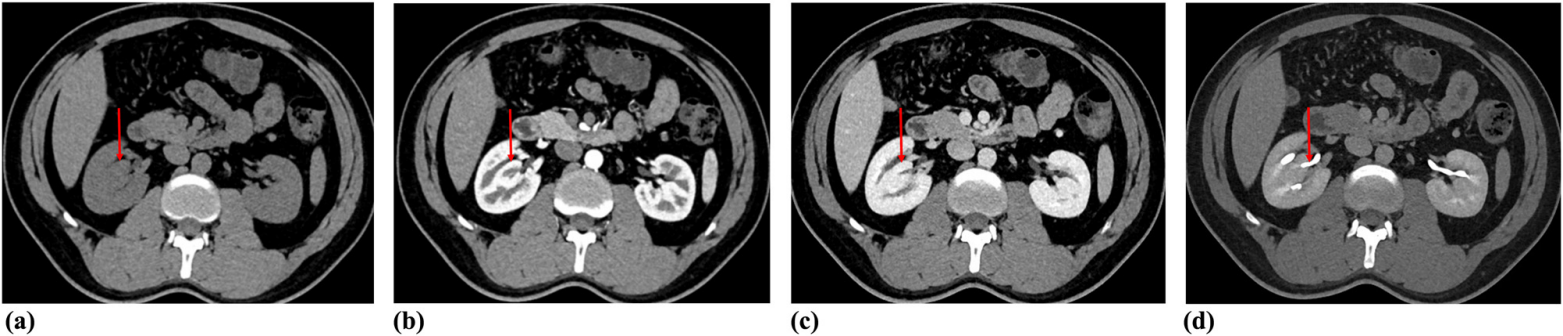
Axial precontrast (a) and contrast-enhanced (b and c) CT showed oval solid mass (red arrows) in right renal sinus with identical density and enhancement pattern to adjacent normal renal cortex. In pyelographic phase image (d) exhibited the small renal pelvis (red arrow) from the pseudo-mass merging into the right renal pelvis.

**Figure 3:**
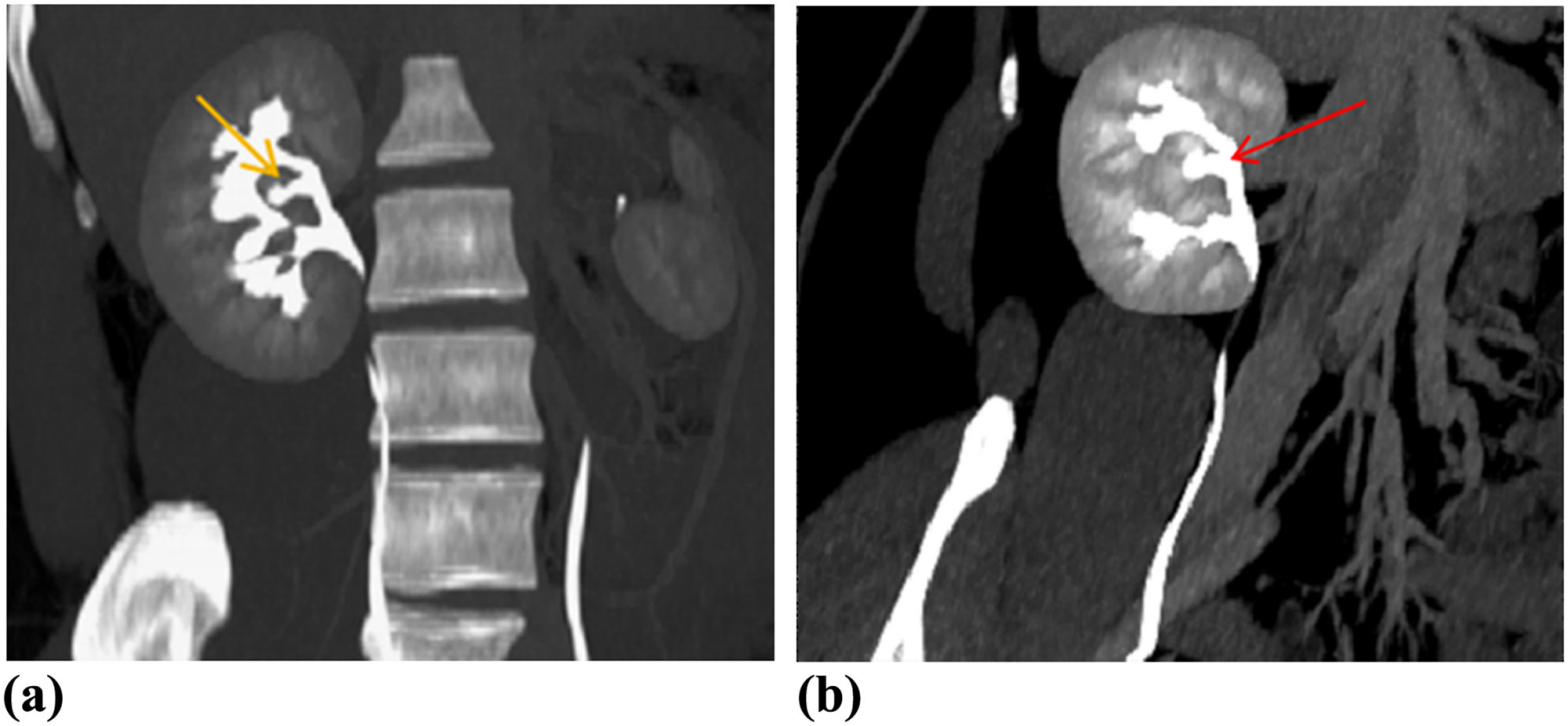
Pyelographic phase coronary MIP (a) and CPR (b) images demonstrated that the little renal pelvis (yellow arrow) from renal shaped mass in the renal sinus drained into the calyces of the upper pole of right kidney (red arrow). MIP = maximum intensity projection; CPR = curved planar reconstruction.


CT urography (CTU) was performed to evaluate the renal lesion more accurately. Plain CT demonstrated an isodensity mass to normal renal parenchyma with unclear boundary in his right renal sinus without hydronephrosis (Figure 2a). The size of lesion was 14 × 5 × 3 mm. Renal contrast-enhanced CT (CECT) showed the intrarenal sinus mass that exhibited the enhancement mode similar to kidney: wheel-spoke shaped enhancement of renal cortex and no enhancement of renal medulla in corticomedullary phase, and homogeneous enhancement in parenchymal phase (Figure 2b and c). Pyelographic phase images demonstrated that a renal pelvis shaped structure from mass merged into the right major renal calyces (Figure 2d). Coronal maximum intensity projection (MIP) CT image in pyelographic phase directly revealed that the mass in renal sinus sent out a renal pelvis shaped structure and merged into the right upper major calyces (Figure 3a). The curved planar reconstruction (CPR) image could display the aberrant duplex kidney and renal pelvis in renal sinuses and the confluence into the right upper renal calices in one plane (Figure 3b).

**Ethics statement:** This study protocol was approved by the Institutional Ethics Committee Board of our hospital. Written informed consent was obtained from the patient for publication of this manuscript and any accompanying images.

## 3 Discussion

Duplex kidney in kidney is very rare. As far as we know, such cases have rarely been reported previously. The duplex kidney in renal sinus and the ipsilateral kidney were integrated without an apparent borderline between them and were supplied by the same ipsilateral renal artery. However, there were two renal pelves that finally drained into the single ureter. No vascular malformation and accessory renal arteries were found in this kind of renal duplication. This disease is very easy to be misdiagnosed because it is asymptomatic or with non-specific symptoms and clinically normal renal function.

The intrarenal duplex kidney is comprised of two separate kidneys and pelvicalyceal systems just like conventional duplex kidney. However, the conventional duplex kidney is frequently associated with duplication of ureter. Moreover, the duplicated renal pelves of conventional duplex kidney generally locate in the upper and lower positions [5]. However, duplex kidney and renal pelvis locates in renal sinus between normal upper and lower major renal calices in this report. Therefore, the intrarenal duplex kidney described in this report may be a rare and special type of embedded type duplex kidney [6].

Intrarenal duplex kidney is usually discovered by accident and closely similar to renal column hypertrophy on ultrasonography. It is usually located in the interior middle portion of the kidney. The key to diagnose intrarenal duplex kidney correctly is the presence of normal renal parenchyma. The key point to diagnose it on imaging is its identical density/signal intensity to renal parenchyma on both precontrast and contrast sequences [5]. To distinguish from renal column hypertrophy, it is critical to identify the duplicated renal pelvis derived from mass within renal sinus draining into the collection system of kidney on pyelographic phase images. Unfortunately, our results lack the pathological diagnosis. Notwithstanding its limitation, our case did demonstrate that the enhancement of mass within renal sinus was similar to background renal parenchyma in arterial and venous phase. The pyelographic phase images and CTU confirmed the diagnosis of intrarenal duplex kidney in our case by showing the duplicated renal pelvis from mass in renal sinus merging into the ipsilateral renal collecting system.

Duplex kidneys are common with the incidence of around 0.8% [1,2]. The majority of duplex kidneys are often of no clinical significance and do not become diseased [4,7,8]. However, almost 50% of the pediatric patients with duplex kidneys have complications requiring treatment [4]. If the duplication of kidney is complicated with renal hypoplasia, atrophy of renal parenchyma, loss of function, ureteral calculi, severe infection or leakage due to ectopic ureteral orifice, ureterocele, hydronephrosis, or vesicoureteral reflux, surgical treatment is needed [9,10]. Heminephrectomy is the most commonly used operation for the management of duplex kidney [11,12,13]. Intrarenal duplex kidneys are a very rare congenital anomaly and take up about 15% of all duplex kidneys [6]. It is important for urologist to get a correct preoperative diagnosis of this type duplex kidney to be more cautious during the heminephrectomy. Conservative treatment remains a challenge due to its very rare occurrence and lack of long-term follow-up data.

In conclusion, intrarenal duplex kidney is prone to misdiagnosed as a renal mass on ultrasonography and plain CT. CECT and CTU are essential for diagnosing it accurately. Prolonged CTU could be helpful in evaluating the duplex kidney with poor function and demonstrating the urinary tract malformations more clearly [8]. If the renal function of the duplex kidney is well, timely diagnosis before operation is crucial to avoid unnecessary radical surgery.

## Abbreviations

CTU: computed tomography urography
CECT: contrast-enhanced CT
MIP: maximum intensity projection
CPR: curved planar reconstruction
